# Traditional Chinese Exercise for Chronic Diseases

**DOI:** 10.1155/2022/9842104

**Published:** 2022-01-25

**Authors:** Xue-Qiang Wang, Li Li, Liye Zou, Kevin W. Chen, Jiao Liu

**Affiliations:** ^1^Department of Sport Rehabilitation, Shanghai University of Sport, Shanghai, China; ^2^Georgia Southern University, Statesboro, USA; ^3^Body-Brain-Mind Laboratory, School of Psychology, Shenzhen University, Shenzhen 518060, China; ^4^Center for Integrative Medicine, University of Maryland School of Medicine, Baltimore, MD, USA; ^5^Fujian University of Traditional Chinese Medicine, Fuzhou, China

Chronic diseases (or noncommunicable diseases) are generally defined as human health conditions that may not have a cure, develop slowly, and require long-term medical management. Major chronic diseases include cardiovascular and cerebrovascular diseases, chronic respiratory diseases, diabetes mellitus, cancer, musculoskeletal disorders, degenerative diseases, and neurological disorders. Of note, these chronic diseases are increasingly recognized as the leading cause of death and disability worldwide and cause a substantial economic burden on healthcare and society.

Physical inactivity has been increasingly recognized as a modifiable risk factor of developing the abovepresented chronic diseases. Conversely, regular physical activity is widely accepted as an easily accessible solution that can maintain health benefits with few side effects, including preventing the onset of chronic diseases or delaying their progression. Traditional Chinese exercises (TCEs) have been utilized as part of traditional Chinese medicine for health promotion and symptomatic management of chronic diseases for thousands of years. TCEs mainly consist of Tai Chi, Baduanjin, Liuzijue, Wuqinxi, and more. These exercises possess a similar philosophy; gentle movements are performed in coordination with muscle stretching and relaxation, slow breathing, proprioceptive awareness (mental focus), and a meditative state of mind. Such unique features have gained increasing popularity worldwide, especially those suffering from chronic diseases with low exercise intolerance. Several interventional studies have shown clinically meaningful improvements associated with TCEs among individuals with Parkinson's disease (PD), fibromyalgia, and heart failure. Further investigations are needed for other chronic diseases such as lower back pain, cancer, chronic obstructive pulmonary disease (COPD), and more. In particular, research on the potential mechanisms of the beneficial effects of TCE is still in its infancy.

In this special issue, we invited researchers to contribute original research, review articles, consensus statements, and guidelines on traditional Chinese exercise in preventing and managing chronic diseases. We were particularly interested in manuscripts that investigated the physiological and psychological responses following TCE intervention among individuals with chronic diseases and their associated biological mechanisms. In total, 17 original and review articles have passed peer review and finally were considered for publication.

A study by M. Shen and others investigated the effects of a 12-week Wuqinxi program on the cognitive measures and motor functions among patients with PD. In this study, 30 PD patients were assigned into two groups (Wuqinxi and stretching training). The two exercise modalities had shared the same intervention duration in which two 90-minute sessions were arranged per week. Results indicated that PD patients demonstrated significant improvement on motor performance following the 12-week Wuqinxi training program. Furthermore, the Wuqinxi group showed significantly better cognitive performance relative to its active control group. Promising findings with small sample size need to be further substantiated.

Similarly, Wang and colleagues attempted to determine the superior effects of Wuqinxi and stretching exercise among 46 mild-to-moderate PD patients. Of note, both acute (one session) and chronic (12 weeks) effects of two exercise modalities on health outcomes were investigated. Interestingly, hand dexterity was significantly enhanced even following one Wuqinxi session. Furthermore, significant improvements on hand dexterity, mental function, and emotional well-being were observed following the 12-week Wuqinxi training. Given the physical and psychological benefits of Wuqinxi training, this unique training approach should be incorporated into part of PD treatment.

X. Yu and others evaluated the effects of Tai Chi in patients with PD through a systematic review and meta-analysis. The study included 17 RCTs with 951 participants. The results suggested Tai Chi is a relatively safe activity for patients with PD to significantly improve general motor function, bradykinesia, and balance conditions but not for their quality of life and functional mobility. The study indicates that more studies with larger sample sizes and better methodological quality are needed to provide more evidence.

X. Yu and the group investigated the effects of body weight support-Tai Chi (BWS-TC) exercise on balance control and walking function in poststroke survivors with hemiplegia. The trial divided 71 subjects into BWS-TC and control groups. The subjects in BWS-TC groups participated in 40 min rehabilitation sessions three times a week for 12 weeks. The results demonstrated that the BWS-TC group showed better performance in gait cycle time, step length, step velocity, range of motion of the joints, and dynamic balance than the control group. This study provides preliminary evidence for patients with stroke to practice Tai Chi, improving balance control and walking function.

Another meta-analysis presented by X. Zhang and colleagues included 19 RCTs aiming to explore the effects of Tai Chi on the balance function and exercise capacity among stroke patients. The Tai Chi group showed better outcomes in the Berg balance function scale, standing and walking test, the center of gravity sway area and speed, and the Fugl–Meyer assessment scale. The study indicates that Tai Chi exercise is beneficial to balance functions and exercise capacities for poststroke patients if practiced once or twice or ≥5 times/week with 30 to 60 minutes each time.

The research work by Y. Qi and coauthors recruited 20 patients with chronic complete thoracic spinal cord injury (SCI). It required them to perform five consecutive sets of 16-form Wheelchair Tai Chi (WCTC16). Researchers recorded and analyzed heart rate variability (HRV) of patients before and after the WCTC16. The results indicated that the Tai Chi exercise was beneficial to vagal activity and sympathetic activity for patients with chronic complete thoracic SCI, which allowed patients to get a balanced sympathovagal tone.

The systematic review and meta-analysis by Q. Sun and the group evaluated the enhancement effects of the combined physical-cognitive interventions on memory self-efficacy, objective cognitive function, psychological well-being, and emotion of older adults with subjective cognitive decline (SCD). The study included 11 RCTs involving 1713 participants with SCD. The interventions were physical activity combined with cognitive training. The study suggested that the combined intervention is an effective and safe method for patients with SCD to improve cognitive function and prevent the conversion of SCD to Alzheimer's disease.

The systematic review and meta-analysis by X. Chen and coworkers evaluated the existing RCTs for evidence of Tai Chi and other TCEs rehabilitation effects for chronic heart failure. The study involved 33 RCTs with 2,465 patients. Compared to the routine managements (RMs) alone, TCEs plus RMs improved peak oxygen consumption (VO_2peak_), 6-minute walking distance (6MWD), and Minnesota Living with Heart Failure Questionnaire (MLHFQ). Compared to general exercise, the TCEs group showed superior improvements in MLHFQ but not in VO_2peak_ or 6MWD. The review suggests that TCEs is a safe and highly adherent rehabilitation therapy, which can be considered an alternative to conventional exercises for patients with chronic heart failure.

Y. Teng and colleagues reviewed 45 original articles regarding the effects of Tai Chi practice on modulating primary hypertension. The review demonstrated the significant efficacy of Tai Chi exercise in improving hypertension clinical symptoms and quality of life, compared to most of the control interventions. Meanwhile, the study also points out the methodological problems in included studies and suggests more future studies with better quality control.

The other systematic review and meta-analysis by C. Kuo and colleagues included 14 RCTs to explore the clinical effects of the Baduanjin exercise among cancer patients. A total of 10 studies were included in the meta-analysis. The meta-analysis supported that the Baduanjin exercise can alleviate the degree of cancer-related fatigue in patients and improve their quality of life and sleep quality, thereby providing preliminary evidence for further long-term follow-up RCTs.

Another research article by C. Yao and coworkers investigated the effect of 24-week Wuqinxi exercise among 72 patients with chronic low back pain (CLBP) through a randomized controlled trial (RCT). All patients who received the Wuqinxi training or general exercise showed improved pain condition and quality of life at the end of 12 weeks and 24 weeks. The Wuqinxi group showed better outcomes than the general exercise group after 24 weeks of intervention. The study results indicate that Wuqinxi can be considered a stand-alone therapy and self-management skill among people with CLBP, suitable for long-term practice.

The research article by Z. Zhang and coworkers reported a parallel control experiment involving 76 patients with amphetamine drug dependence, divided into Taijiquan and control groups. The Taijiquan group received a 6-month exercise intervention and the same routine rehabilitation exercise as the control groups. The study reported that Taijiquan exercise was beneficial to the balance control ability, overall sense of health, vitality, mental health, trait anxiety, and drug craving in the dependent patients. The study provided evidence to support exercise therapy for addicts to quit drugs and prevent relapse.

The paper lead by T. Xiao with colleagues used the design of three-arm RCT. It aimed to explore the efficacy of group-based basketball and Baduanjin exercise on problematic smartphone use and mental health in eligible Chinese college students. The study included 96 participants for three groups, basketball, Baduanjin, and control groups. They reported a significant reduction of problematic smartphone use and improved mental health, including feelings of anxiety, loneliness, inadequacy, and perceived stress after 12-week interventions of basketball and Baduanjin, respectively. Furthermore, similar improvements were still significant at two-month follow-up except for perceived stress. The study demonstrates that exercise is helpful for college students with problematic smartphone use and their mental health.

The systematic review by J. Fang and the group included 47 trials involving 3877 participants. The review investigated the reported adverse events potentially associated with Baduanjin exercise and evaluated the quality of the methods used to monitor adverse events in the trials assessed. Only two studies reported adverse events that were potentially caused by Baduanjin exercise, such as muscle ache, palpitation, giddiness, knee pain. The limited evidence suggests the future studies to monitor and record the adverse events strictly.

J. Zhou and coworkers comprehensively reviewed and summarized clinical studies on Baduanjin to provide more evidence-based evidence in support of the application of Baduanjin for healthcare. A total of 810 publications were identified, including 43 systematic reviews, 614 RCTs, 66 non-RCTs, 84 case series, and 3 case reports. The study reported that the most commonly used version of Baduanjin was the style of the State General Administration of Sport of China in 2003. There were no serious adverse events related to the Baduanjin intervention. The reviewers suggested further high-quality designed and reported studies to validate the clinical benefits of Baduanjin.

In their review, Y. Qin and coworkers updated the clinical effects of traditional Chinese exercises, such as Tai Chi, Baduanjin, Yijinjing, and Wuqinxi, in the treatment of simple obesity. The review showed beneficial clinical effects on the treatment of simple obesity with their characteristic. For example, Tai Chi is beneficial to the overall balance, while Baduanjin can increase lower limb strength and reduce fat content. Thus, the review suggests that traditional Chinese exercises can reduce fat and weight, adjust posture, and cultivate physical and mental health, which is a simple and effective way to lose weight at low cost and is environment friendly.

The protocol reported by T. Liu and the group aimed to explore the specific and nonspecific effects of Tai Chi and its responses in patients with functional constipation. The trial will recruit 40 patients with functional constipation and 40 healthy subjects and require them to receive a 10-week Tai Chi intervention. The outcomes such as motor function, respiratory function, emotional state, 24 h dynamic electrocardiogram, and the functional magnetic resonance imaging will be evaluated at the baseline, after 5-and 10-week Tai Chi practice. This study provides a new perspective for understanding Tai Chi and a new approach for investigating the mind-body exercise.

We hope that readers will be interested in understanding the physiological and psychological effects and the underlying mechanisms of traditional Chinese exercise for chronic diseases, such as stroke, SCI, PD, and chronic low back pain. We also hope the special issue can perk the interest of more researchers studying the effects of traditional Chinese exercise for chronic diseases, thereby contributing and improving further investigations and research studies with new strategies and high-quality design.

*Xue-Qiang Wang* *Li Li* *Liye Zou* *Kevin Chen* *Jiao Liu*

